# TNFα promotes CAR-dependent migration of leukocytes across epithelial monolayers

**DOI:** 10.1038/srep26321

**Published:** 2016-05-19

**Authors:** Penny E. Morton, Alexander Hicks, Elena Ortiz-Zapater, Swetavalli Raghavan, Rosemary Pike, Alistair Noble, Abigail Woodfin, Gisli Jenkins, Emma Rayner, George Santis, Maddy Parsons

**Affiliations:** 1 Randall Division of Cell and Molecular Biophysics, King’s College London, New Hunt’s House, Guys Campus, London, SE1 1UL, UK; 2 Division of Asthma, Allergy & Lung Biology, King’s College London, 5th Floor Tower Wing, Guy’s Hospital Campus, London, SE1 1UL, UK; 3 William Harvey Research Institute, Barts and the London School of Medicine and Dentistry, Queen Mary, University of London, EC1M 6BQ, UK.; 4 Respiratory Research Unit, University of Nottingham, Clinical Sciences Building, City Campus, Nottingham, NG5 1PB, UK; 5 Public Health England, Salisbury, Wiltshire, SP4 0JG, UK

## Abstract

Trans-epithelial migration (TEpM) of leukocytes during inflammation requires engagement with receptors expressed on the basolateral surface of the epithelium. One such receptor is Coxsackie and Adenovirus Receptor (CAR) that binds to Junctional Adhesion Molecule-like (JAM-L) expressed on leukocytes. Here we provide the first evidence that efficient TEpM of monocyte-derived THP-1 cells requires and is controlled by phosphorylation of CAR. We show that TNFα acts in a paracrine manner on epithelial cells via a TNFR1-PI3K-PKCδ pathway leading to CAR phosphorylation and subsequent transmigration across cell junctions. Moreover, we show that CAR is hyper-phosphorylated *in vivo* in acute and chronic lung inflammation models and this response is required to facilitate immune cell recruitment. This represents a novel mechanism of feedback between leukocytes and epithelial cells during TEpM and may be important in controlling responses to pro-inflammatory cytokines in pathological settings.

The epithelium plays a complex and active role in a wide range of biological processes such as embryonic development, tissue homeostasis, wound healing and inflammation^1^,^2^. The migration of leukocytes across the epithelium is critical to elicit efficient tissue immune responses. Transmigration is a complex multi-step process that requires multiple interactions between transmembrane proteins on both leukocytes and epithelial cells to enable adhesion, transmigration and release into the luminal space^3^,^4^. Tight and adherens junctions are responsible for maintaining epithelial integrity by providing a link between adjacent epithelial cells; they are also important for interactions with immune cells and regulate their controlled passage across the epithelium^5^,^6^,^7^. Indeed, disruption of these cell-cell junctions has been associated with inflammatory diseases such as asthma^8^.

Coxsackie and Adenovirus (Ad) Receptor (CAR) was initially identified as the primary docking receptor for Coxsackie B viruses and members of the Ad family^9^. Further work has since demonstrated that CAR is an important cell adhesion molecule^10^,^11^ and a member of the Junction Adhesion Molecule (JAM) family that forms homo-dimers across cell-cell junctions^12^,^13^. We have recently shown that CAR is phosphorylated at S290/T293 by PKCδ and this controls E-Cadherin stability at adherens junctions^14^,^15^. This function of CAR appears to be tissue-specific as CAR knockout mice have defects in lymphatic vessels^16^, lung and heart but not within the intestinal epithelium^17^. CAR has also been shown to bind to another of the JAM family proteins, JAM-L, that is expressed on the surface of leukocytes and this facilitates leukocyte transmigration and activation^3^,^18^,^19^,^20^. However, it remains unclear whether CAR:JAM-L triggers changes in the epithelial cell junction to facilitate TEpM, and if so, what external cues are responsible for initiating these changes.

Here, we have tested our hypothesis that CAR plays an active role in TEpM of leukocytes under inflammatory conditions. Our data demonstrates that phosphorylation of the cytoplasmic tail of CAR is required for efficient leukocyte TEpM and that CAR is phosphorylated both *in vitro* and *in vivo* specifically in response to TNFα and through a PI3K and PKCδ pathway. This data reveals a key novel role for CAR in leukocyte TEpM in inflammatory conditions.

## Results

### Phosphorylation of CAR is required for transmigration of THP-1 cells

**Figure 1:**
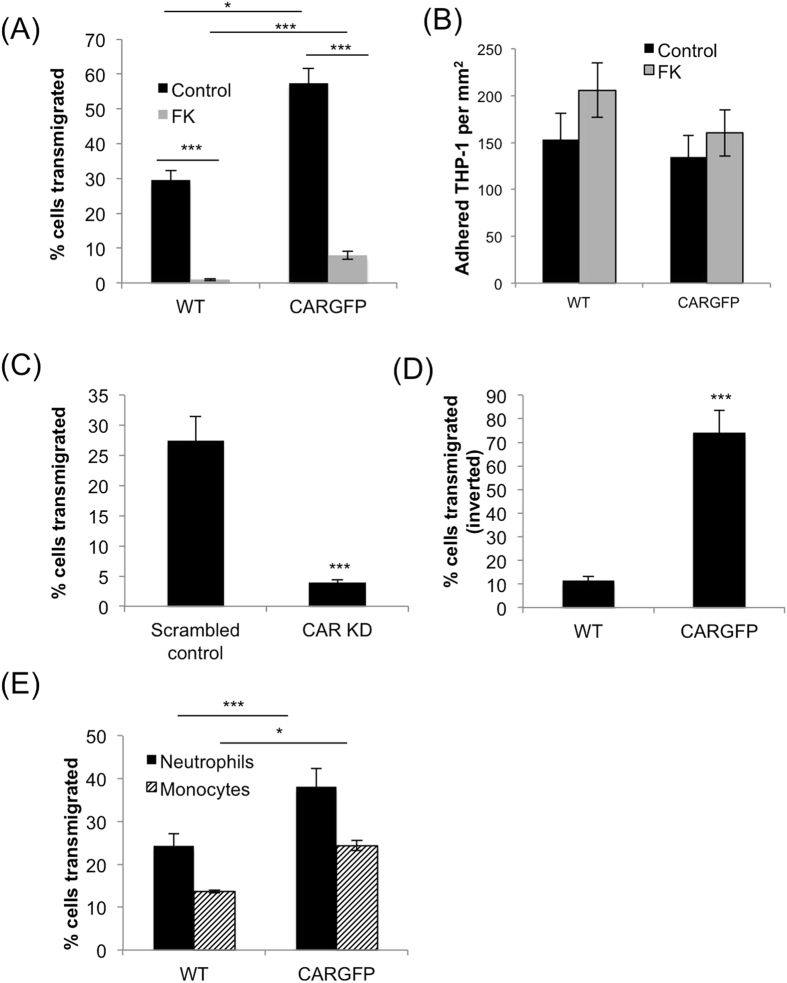
CAR promotes transmigration of leukocytes across human bronchial epithelial cell monolayers. (**A**) THP-1 cells were stained using cell-tracker orange before adding to the top well of transwell inserts containing WT or CAR-GFP HBEC monolayers and pre-treated with BSA (control) or recombinant fibre knob protein (FK). THP-1 cells in the bottom well were counted after 24 hours using FACS. Data is presented as relative fold change of THP-1 cell number transmigration (TEM) compared to WT control conditions from 4 independent experiments + /−SEM. (**B**) Quantification of THP-1 adhesion to WT or CAR-GFP HBEC. Experiments were set-up as in (**A**) and number of adhered THP-1 cells to the HBEC monolayer assessed after 24 hours. Data is representative of four independent experiments; mean values from n = 4 wells + /−SEM. (**C**) THP-1 cells were labelled using cell-tracker orange before adding to the top of transwell inserts containing monolayers of 16HBE14-o cells stably expressing control (scrambled) or CAR-targeted shRNA. THP-1 cells in the bottom well were counted after 24 hours using FACS. Data is presented as relative fold change of THP-1 cell number compared to WT control conditions from 4 independent experiments + /−SEM. **(D**) THP-1 cells were added to the underside of transwell inserts containing monolayers of WT or CAR-GFP-HBEC and allowed to adhere before inserts were inverted for 8 hours in the presence of a serum gradient to allow baso-lateral to apical migration of THP-1 cells. Data is representative of four independent experiments and presented as relative fold change of THP-1 cell number compared to WT control conditions from 4 wells + /−SEM. (**E**) Transmigration of primary human neutrophils and monocytes was analysed using Transwell chambers containing monolayers of WT and CAR-GFP-HBEC as in (**A**). Data is presented as relative fold change of THP-1 cell number compared to WT control conditions from 4 independent experiments + /−SEM.

CAR has previously been shown to be a receptor for JAM-L that is expressed on opposing membranes of leukocytes and required for transmigration across cell monolayers^18^. To test the hypothesis that phosphorylation of CAR may be involved in leukocyte transmigration, THP-1 cells (that are known to express JAM-L;^21^) were incubated with control or CAR-GFP over-expressing human bronchial epithelial cells (HBEC) that we have previously characterised^15^ and left to transmigrate for 48 hours. Overexpression of CAR-GFP significantly increased THP-1 cell transmigration through, but not adhesion to, the epithelial layer (Fig. 1A,B). Pre-incubation of cells with recombinant Ad5 fibre knob (FK) which binds to CAR with higher affinity than CAR binds to itself (Kirby *et al*.^22^), inhibited THP-1 transmigration but had no effect on THP-1 cell adhesion to the epithelial monolayer (Fig. 1A,B). This data suggests that THP-1 cell migration, but not adhesion might be dependent on the ability of CAR to homo-dimerise in *trans*. Alternatively, as the binding sites on CAR for JAM-L and FK overlap^18^,^20^,^22^, FK may inhibit THP-1 transmigration by competitively blocking THP-1 cells binding to CAR at cell-cell junctions.

HBEC express low levels of endogenous CAR^15^, so we additionally tested the role of CAR in TEpM using an alternative well-characterised human bronchial epithelial cell line, 16HBE14-o. These cells express relatively high endogenous levels of CAR compared to HBEC and were therefore amenable to shRNA-mediated CAR knockdown (Supp Fig. 1A). In support of our findings in HBEC, THP-1 cell transmigration was significantly reduced through monolayers of 16HBE14-o CAR knockdown cells (Fig. 1C). Leukocytes migrate through epithelia in a basal to apical direction therefore we analysed CAR dependent TEpM using a more physiological inverted transmigration assay where THP-1 adhere to the basal surface of the epithelial monolayer before migrating towards the apical surface. Figure 1D shows that expression of CAR-GFP significantly increased the number of THP-1 able to fully migrate to the apical surface of the HBEC monolayer (Fig. 1D) compared with wild-type (WT) cells. Finally, we tested whether CAR could also facilitate TEpM of primary monocytes and neutrophils isolated from human blood, both of which express high levels of JAM-L^23^. Data demonstrated that both cell types showed significantly higher transmigration through HBEC overexpressing CAR-GFP compared to WT cells (Fig. 1E) in agreement with our THP-1 cell line data. These data collectively demonstrate that monocytic cell binding to epithelial cells is facilitated through CAR and that this association is required for efficient trans-epithelial migration.

### THP-1 cells stimulate CAR phosphorylation, which is required for transmigration

**Figure 2:**
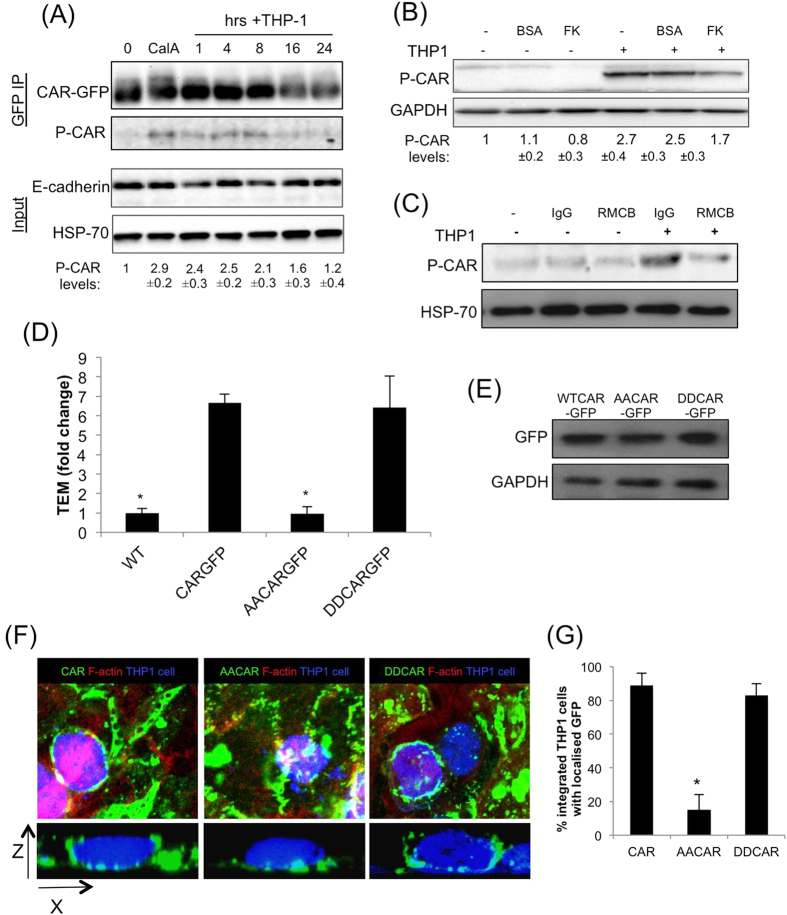
CAR promotes THP-1 transmigration in a phosphorylation dependent manner. (**A**) THP-1 cells were added to CAR-GFP HBEC monolayers for the times indicated. Cells were then lysed and immunoprecipitated with anti-GFP antibodies followed by western blotting using antibodies against p-CAR, and GFP. Total cell lysates were probed for E-cadherin and HSP-70 as loading controls. Calyculin A (CalA) treatment was used as a positive control to induce p-CAR. Values beneath blots are relative levels of p-CAR compared to time 0 from 4 independent experiments + /−SEM. (**B**) Western blot of phosphorylated CAR as in (**A**) following 4 h incubation with THP-1 cells with or without pre-treatment with 100 μg/ml FK or BSA as a control. Values beneath blots are relative levels of p-CAR compared to untreated from 4 independent experiments + /−SEM. (**C**) Western blot of phosphorylated CAR as in (**A**) following 4 h incubation with THP-1 cells with or without pre-treatment with 10 μg/ml RMCB CAR function blocking antibody or IgG as a control. Values beneath blots are relative levels of p-CAR compared to untreated from 4 independent experiments + /−SEM. (**D**) THP-1 transmigration analysis performed as in (**A**) using WT, CAR-GFP, AACAR-GFP and DDCAR-GFP HBEC. Error bars are SEM. Statistical significance compared with CAR-GFP HBEC is shown. (**E**) Representative western blot of levels of CAR-GFP in specified cells lines. (**F**) Example confocal images of THP-1 cells undergoing trans-epithelial migration. Cell tracker orange stained THP-1 cells were added to confluent monolayers of HBEC and fixed after 24 hours before confocal imaging to obtain z-stacks. Cell tracker orange THP-1 cells (blue), CAR-GFP (green) and actin (red) to show the position of the HBEC are shown. Z-slice and maximum intensity projection show the location of THP-1 in relation to HBEC monolayer and recruitment of CAR-GFP to THP-1. (**G**) Quantification of % of cells with lateral associated CAR-GFP in images as from (**F**) Data is mean of 18 fields of view over 3 independent experiments. For all data where relevant * = p < 0.005 compared to CAR-GFP.

Our recent discovery that CAR can be phosphorylated at cell-cell junctions^15^ prompted us to examine CAR phosphorylation status during THP-1 integration and transmigration. THP-1 cell interactions with CAR-GFP HBEC led to a prolonged increase in phospho-CAR that peaked at around 4–8 h post THP-1 addition (Fig. 2A), and this peak correlated with the time taken for THP-1 to fully integrate into HBEC monolayers. The p-CAR signal we detected was specific to CAR expressed in HBEC as THP1 cells do not express detectable levels of CAR (data not shown). Treatment of CAR-GFP cells with soluble JAML protein did not induce CAR phosphorylation, implying that CAR ligation alone is not sufficient for this activation step (data not shown). THP-1 cell-induced CAR phosphorylation was inhibited by pre-treatment of cells with FK (Fig. 2B) or function blocking CAR antibody RMCB (Fig. 2C) further demonstrating that THP-1-CAR binding was required for CAR phosphorylation to occur. Antibody-induced crosslinking of CAR-GFP HBEC was not able to induce CAR phosphorylation (data not shown), further suggesting that the presence of THP-1 cells and potentially other signals are required for phosphorylation to occur.

To investigate whether the observed increase in phospho-CAR played a role in TEpM, we analysed transmigration across HBEC expressing phospho-mimic (DDCAR-GFP) or phospho-dead (AACAR-GFP) CAR-GFP that localise similarly to cell-cell junctions as compared to CAR-GFP (Supp Fig. 1B;^15^). Data demonstrated that expression of DDCAR-GFP but not AACAR-GFP supported enhanced THP-1 transmigration across HBEC despite equal levels of CAR-GFP being expressed in these cell lines (Fig. 2D,E). Expression of AACAR-GFP and DDCAR-GFP had no effect on epithelial monolayer permeability under unstimulated conditions compared to wild-type HBEC or those expressing CAR-GFP (Supp Fig. 1C). Expression of another key surface receptor molecule involved in leukocyte transmigration, Intercellular Adhesion Molecule-1 (ICAM1), was unchanged upon CAR overexpression in 16HBE cells or treatment with TNFα (Supp Fig. 1D). However, ICAM1 levels were slightly elevated in HBEC expressing AACAR compared to those expressing FLCAR (Supp Fig. 1D). The mechanisms or consequences of the increased ICAM1 expression specifically in phospho-dead expressing CAR cells remains unknown, but given that ICAM1 is a positive regulator of TEM, this data strongly implies that changes in ICAM1 do not contribute to altered TEM seen in cells overexpressing CAR. Confocal analysis and quantitation further revealed that CAR-GFP and DDCAR-GFP but not AACAR-GFP was clustered around transmigrating THP-1 cells (Fig. 2F,G). Combined, this data demonstrates that CAR is required for efficient transmigration of leukocytes and this is promoted by phosphorylation of the CAR cytoplasmic tail.

### TNFα stimulates CAR phosphorylation via PI-3-K and PKCδ

**Figure 3:**
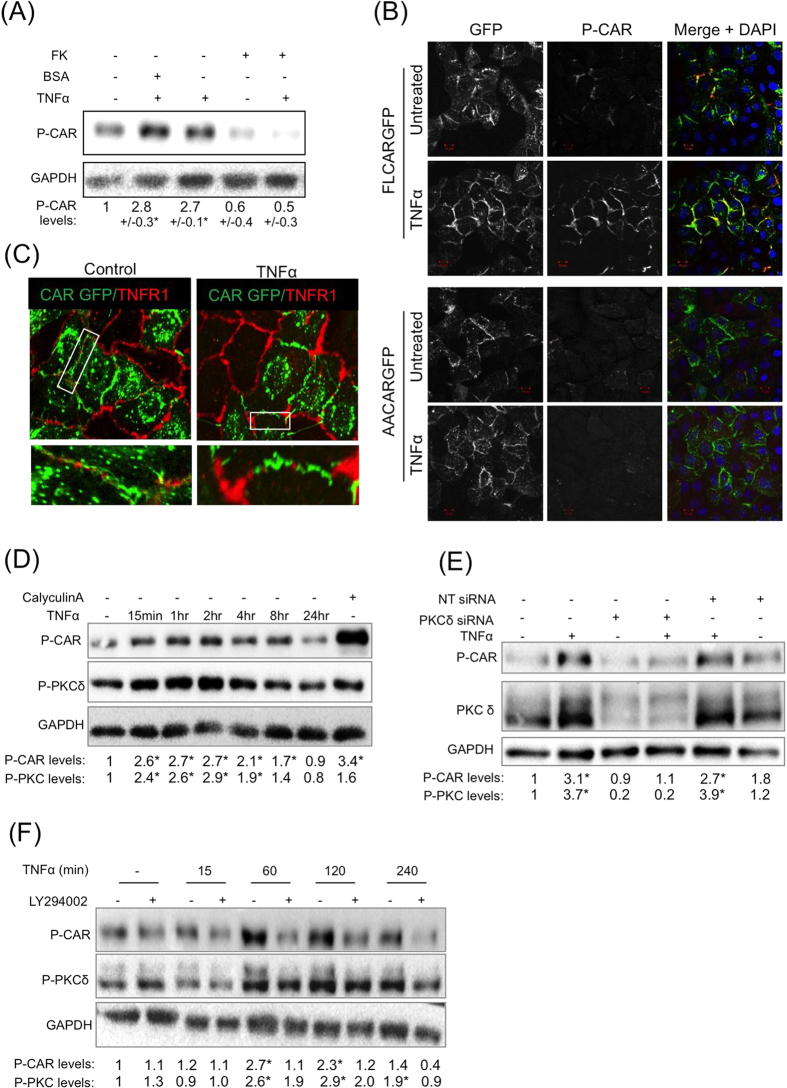
TNFα stimulates CAR phosphorylation downstream of P-I-3K and PKCδ. (**A**) Western blot analysis of CAR-GFP HBEC treated with 10 ng/ml TNFα (60 min) and 200 μg/ml FK (120 min) or BSA control as indicated, probed for phospho-CAR and GAPDH as a loading control. Values beneath blots are relative levels of p-CAR compared to time 0 from 4 independent experiments + /−SEM. (**B**) Confocal images of CAR-GFP HBEC or AACAR-GFP HBEC mixed 1:1 with WT HBEC treated with 10 ng/ml TNFα and immunostained for phospho-CAR. Arrows indicate increased phospho-CAR at junctions following treatment with TNFα. Images are maximum intensity projections of z-stacks through the entire z-depth of the cells acquired using identical Z-depth acquisition settings. Scale bars are 10 μm. (**C**) Confocal images of endogenous TNFR1 (red) localisation in a 1:1 WT:CAR-GFP (green) HBEC mix untreated cells or treated with 10 ng/ml TNFα for 60 min. White boxes are highlighted inset panels below. (**D**) Western blot analysis of phospho-CAR and phospho-PKCδ after treatment with 10 ng/ml TNFα for the indicated times. Western blots were also probed for GAPDH as a loading control. Calyculin A was used as a positive control. Values beneath blots are relative levels of p-CAR compared to untreated from 4 independent experiments + /−SEM. * = p < 0.01. (**E**) Western blot analysis of phospho-CAR in CAR-GFP HBEC transfected with PKCδ siRNA or non-targeting control (NT). Cells were treated with 10 ng/ml TNFα for 60 min where indicated. Western blots were probed for phospho-CAR, PKCδ and GAPDH as a loading control. Values beneath blots are relative levels of p-CAR compared to untreated NT siRNA from 3 independent experiments + /−SEM. (**F**) Western blot analysis of phospho-CAR and phospho-PKCδ after pre-treatment with 10 μM LY294002 for 2 hours where indicated and further treatment with 10 ng/ml TNFα for the times shown. Western blots were also probed for GAPDH as a loading control. Values beneath blots are relative levels of p-CAR compared to untreated from 3 independent experiments + /−SEM.

Pro-inflammatory cytokines play an important role in mediation of leukocyte transmigration^24^ and have been shown to regulate JAM family protein localisation and function^25^,^26^,^27^,^28^. We therefore hypothesised that CAR phosphorylation may be driven by a cytokine-induced signalling pathway. Treatment of cells with a panel of pro-inflammatory cytokines TNFα, IL-5, IL-1β, IL-13 and IL-17^25^,^27^,^28^,^29^ revealed that TNFα treatment induced a robust increase in phospho-CAR in CAR-GFP but not AACAR-GFP expressing HBEC (Fig. 3A). TNFα treatment also induced significant phosphorylation of endogenous CAR in 16HBE cells (Supp Fig. 1E). Moreover, TNFα-induced phosphorylation of CAR was inhibited by pre-treatment of cells with Ad5FK suggesting that CAR homo-dimerisation was required for this response to stimulation (Fig. 3A). Confocal analysis further revealed that TNFα-induced CAR phosphorylation occurred predominantly at cell-cell junctions supporting the notion that homo-dimerised CAR *in trans* is required for CAR phosphorylation downstream of TNFα (Fig. 3B). IL-5 also induced modest CAR phosphorylation but to a much lesser extent compared with TNFα (Supp Fig. 1F). None of the other cytokines tested induced CAR phosphorylation (Supp Fig. 1G). We therefore explored the co-operation between TNFα and CAR in more detail.

TNFα signals predominantly through TNF receptor1 (TNFR1) in epithelial cells, so we postulated that CAR and TNFR1 might be able to form a complex at the membrane in the presence of inflammatory cues. However, confocal imaging revealed that whilst both TNFR1 and CAR were present at CAR-GFP HBEC cell-cell junctions and CAR-GFP expression did not affect TNFR1 protein levels (Supp Fig. 1H), the two receptors did not show significant colocalisation (Fig. 3C) suggesting that CAR may potentially relocalise distribution of TNFR1 within the plasma membrane. We have previously shown that CAR is phosphorylated by PKCδ^15^ and this kinase is also activated downstream of TNFα-TNFR1 binding and PI-3-kinase (PI-3-K) in neutrophils^18^. To determine whether this pathway was important in control of CAR phosphorylation, we analysed levels of active PKCδ in the same samples as p-CAR following TNFα treatment. Western blot analysis demonstrated that TNFα induced PKCδ activity in HBEC and this correlated with the time-course of CAR phosphorylation in the same samples (Fig. 3D). Moreover, siRNA mediated depletion of PKCδ or inhibition of PI-3-K by treatment with LY294002 both inhibited TNFα-induced CAR phosphorylation (Fig. 3E,F) supporting a role for this pathway in CAR-dependent induction of TEpM.

### TNFα induced CAR phosphorylation promotes TEpM of THP-1 cells

**Figure 4:**
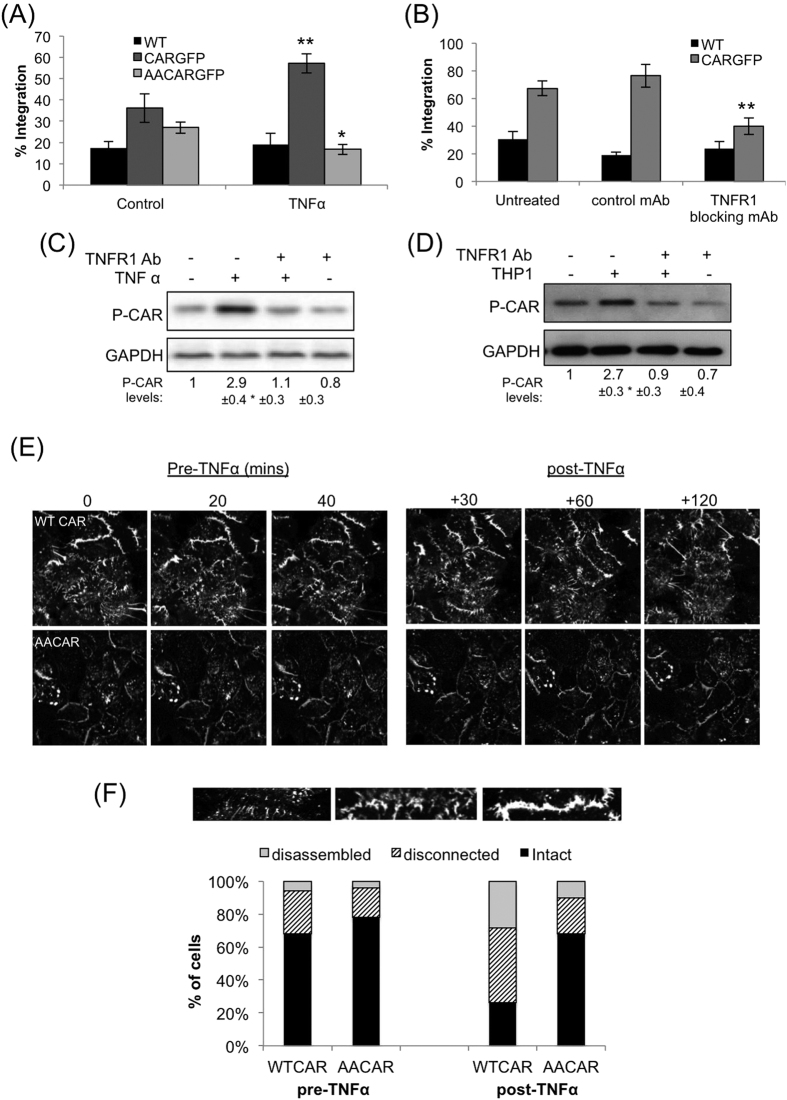
TNFα promotes THP-1 integration into epithelial layers in a phospho-CAR dependent manner. (**A**) Analysis of THP-1 integration into WT, CAR-GFP or AACAR-GFP HBEC monolayers 4 hours after addition of THP-1. Where indicated, HBEC cultures were pre-treated with 10 ng/ml TNFα for 1 hour prior to addition of THP-1. (**B**) Analysis of THP-1 integration into WT, CAR-GFP or AACAR-GFP HBEC monolayers 6 hours after addition of THP-1. Where indicated, HBEC cultures were pre-treated with 10 μg/ml anti-TNFR1 blocking antibody or an isotype specific control antibody, for 1 hour prior to addition of THP-1. For both A and B, statistical significance compared with untreated control is shown. (**C**) Western blot analysis of phospho-CAR in CAR-GFP HBEC treated with 10 ng/ml TNFR1 blocking antibody or a control antibody, and further treated with 10 ng/ml TNFα where indicated. GADPH was used as a loading control. (**D**) Western blot analysis of phospho-CAR in CAR-GFP HBEC cultured with THP-1 cells for 4 hours. Where indicated, CAR-GFP HBEC were treated with 10 μg/ml TNFR1 blocking antibody prior to addition of THP-1. GADPH was used as a loading control. Values beneath blots are relative levels of p-CAR compared to untreated from 4 independent experiments + /−SEM. (**E**) Confocal time-lapse microscopy analysis of junction dynamics in CAR-GFP and AACAR-GFP HBEC prior and post addition of 10 ng/ml TNFα as indicated above images. Images are stills taken from the time-lapse movies. (**F**) Analysis of cell-cell junctions in images taken as in (**E**) CAR-GFP and AACAR-GFP HBEC positive junctions were characterised as shown and presented as percentage of the total; n = 90 junctions per condition over 3 different experiments. Where relevant, * = p < 0.05, ** = p < 0.01 *** = p < 0.005.

TNFα is known to affect the stability and structure of endothelial cell junctions to promote trans-endothelial migration of leukocytes during inflammation^24^,^26^. We therefore postulated that TNFα could promote TEpM of leukocytes through phosphorylation of CAR. We first sought to clarify whether TNFα could promote integration of leukocytes within epithelial monolayers, and whether this was CAR-dependent. Our analysis revealed that CAR-GFP HBEC monolayers stimulated with 10 ng/ml TNFα showed significantly higher THP-1 integration after 4 hours than untreated monolayers (Fig. 4A). Moreover, WT HBEC (that express very low levels of CAR) and AACAR-GFP-HBEC did not exhibit an increase in THP-1 integration when treated with TNFα (Fig. 4A). Thus phosphorylation of CAR is required for TNFα-induced transmigration of THP-1 cells.

THP-1 cells have previously been shown to secrete TNFα^30^ so we next investigated whether THP-1 may act in a paracrine fashion to promote transmigration through HBEC. Pre-incubation of CAR-GFP HBEC with TNFR1 blocking antibodies, at a concentration that completely blocked TNFα induced TNFR1 activation (Supp Fig. 1I), resulted in reduced THP-1 integration into CAR-GFP HBEC monolayers (Fig. 4B) thus confirming a requirement for TNFR1 in this process. Moreover, blocking TNFR1 function also inhibited both TNFα- and THP-1 induced CAR phosphorylation (Fig. 4C,D) further demonstrating a requirement for TNF-TNFR1 engagement in controlling CAR phosphorylation. Taken together these data show that THP-1 cells induce CAR phosphorylation in epithelial cells via TNFR1 to promote their integration.

We next sought to understand whether CAR phosphorylation and TNFα might facilitate increased THP-1 TEpM by analysing the stability of junction dynamics in HBEC expressing CAR-GFP and AACAR-GFP. Time-lapse confocal imaging of HBEC revealed that in the absence of stimulation, CAR-GFP and AACAR-GFP positive cell-cell junctions exhibited similar predominantly stable cell-cell contacts (Fig. 4E, quantified in F). However after addition of TNFα, CAR-GFP positive junctions became more dynamic, exhibiting increased instability, discontinuity and movement of cell-cell interfaces within the monolayer (Fig. 4E, top left panels). In contrast, AACAR-GFP positive junctions showed minimal change in dynamic behaviour following TNFα stimulation (Fig. 4E, bottom left panels, and 4F). Moreover, analysis of FITC-dextran permeability assays over the same time periods of TNF treatment demonstrated a significant increase in CAR-GFP cell permeability but not in AACAR-GFP expressing cells (Supp Fig. 1J). These data support a role for CAR phosphorylation in the control of cell-cell junction dynamics.

### Inflammatory signals trigger CAR phosphorylation and leukocyte recruitment to the bronchial epithelium

**Figure 5:**
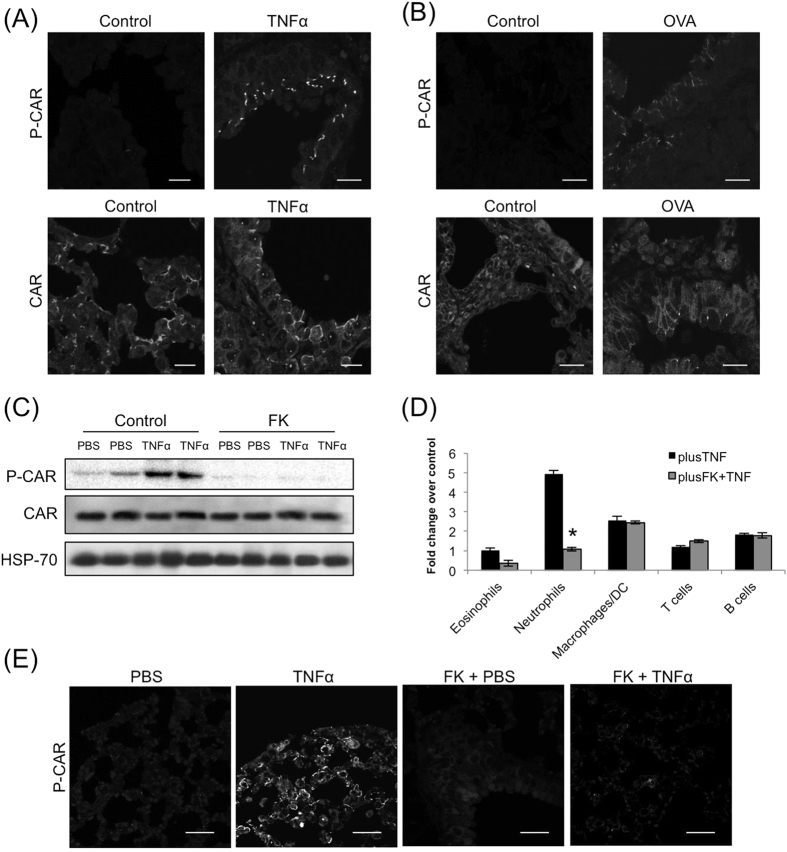
TNFα promotes trans-epithelial migration of leukocytes in a CAR dependent manner *in vivo.* (**A**) Confocal analysis of fixed lung sections from C57BL/6 (B6) mice sensitized with 1 μg recombinant murine TNFα in 50 μl of PBS or PBS alone and immunostained separately for phospho-CAR (top) or total CAR (bottom). (**B**) Confocal analysis of lung sections from Ovalbumin stimulated (OVA) or PBS treated control mice. Sections were stained with phospho-CAR (top) or total CAR (bottom). (**C**) Western blot analysis of phospho-CAR in whole lung lysates from of C57BL/6 (B6) mice sensitized with 1 μg recombinant murine TNFα and total CAR alone or pre-sensitized with 500 μg FK as indicated. HSP-70 was used as a positive control. (**D**) BAL data from C57BL/6 (B6) mice sensitized with 1 μg recombinant murine TNFα alone or pre-sensitized with 500 μg FK as indicated. Data is pooled from four different mice + /−SEM and representative of two experiments. * = p < 0.001 compared to TNFα alone. **(E)** Confocal analysis of lung sections from experiments shown in C and D immunostained with phospho-CAR antibodies. Scale bars correspond to 20 μm.

TNFα is known to promote leukocyte trans-endothelial migration^26^ as well as epithelial monolayer stability (Coyne *et al*.^5^) and may therefore function as key mediator of CAR function during inflammation *in vivo*. We addressed this hypothesis using two different mouse models of lung inflammation. Firstly, we assessed levels of CAR phosphorylation in a murine model of TNFα-induced acute lung inflammation. Analysis of sections of lung epithelium following intra-tracheal TNFα challenge for 24 hours resulted in an increase in CAR phosphorylation at junctions between small airway epithelial cells without affecting total CAR levels or localisation (Fig. 5A) as well a recruitment of immune cells to the bronchial epithelium (Supp Fig. 2A). Secondly, we analysed CAR phosphorylation in an ovalbumin-induced chronic lung inflammation model. Under these conditions, chronic inflammation also induced CAR phosphorylation, specifically at cell-cell junctions of small bronchioles (Fig. 5B) as well a recruitment of immune cells to the bronchial epithelium (Supp Fig. 2B). Importantly, repeating the same challenge in TNFR knockout mice resulted in no effect on CAR phosphorylation, suggesting that TNF can trigger CAR phosphorylation via a TNFR1-driven pathway *in vivo* (Fig. S2C,D). This data shows that CAR phosphorylation and neutrophil recruitment into the broncho-alveolar spaces occur over a similar time frame during inflammation. Moreover, exogenously applied TNFα stimulates CAR phosphorylation and promotes migration of leukocytes in the bronchial lumen. This would suggest that heightened TNFα levels such as those found during inflammation might drive CAR-dependent immune cell integration into epithelial tissues *in vivo*.

Our data showed that pre-incubation of CAR-GFP HBEC cells with FK successfully blocked TNFα mediated CAR phosphorylation (Fig. 3B). In order to investigate whether this may also be the case *in vivo*, FK was delivered into mice via intra-tracheal administration followed by challenge with TNFα. Western blot and immunohistochemistry analysis revealed that administration of FK significantly reduced CAR phosphorylation both in whole lungs (Fig. 5C) and lung sections (Fig. 5E) respectively. Moreover, bronchial lavage of these mice showed significant TNFα-induced bronchial egression of neutrophils (Fig. 5D), which have previously been shown to utilise CAR:JAM-L interactions to undergo TEpM^3^. Importantly FK also blocked TNFα-stimulated neutrophil egression, showing that TNFα stimulated neutrophil migration into the bronchial lumen is dependent on phosphorylated CAR in bronchial epithelial cells. Interestingly, in haematoxylin and eosin stained lung sections from the same mice we observed that FK did not decrease general recruitment of immune cells to the bronchiolar epithelium (Supp Fig. 2E). This indicates that the effect of inhibiting CAR phosphorylation may be specific to sub-sets of immune cells, possibly those expressing JAM-L such as neutrophils. Taken together these data demonstrate the importance of inflammation-induced CAR phosphorylation *in vivo*.

## Discussion

In this study we show that *in trans* homo-dimers of CAR within epithelial cell-cell adhesions are phosphorylated in response to exogenous TNFα and that this contributes to efficient transmigration of monocytes and neutrophils both *in vitro* and *in vivo*. Our data further suggests that CAR may be a novel important receptor in the control of inflammation downstream of TNFR1-dependent signalling and thus may represent an alternative therapeutic target in control of chronic inflammatory lung disease.

The data we present here shows that CAR is important in transmigration of leukocytes, but not initial adhesion of these cells to the epithelial monolayer. CAR has been shown to be present in both tight and adherens junctions, but is low or absent at the apical surface of polarised cell monolayers^10^,^14^,^31^,^32^. This lack of available CAR at the apical surface for JAM-L binding on leukocytes would suggest alternative adhesion molecules are involved in the initial leukocyte adhesion and tethering steps to the cell monolayer. Indeed, a number of epithelially expressed adhesion receptors including integrins and selectin family members have been shown to participate in this process during inflammation^33^. Following adhesion, the leukocytes are then able to access CAR at tight and adherens junctions through JAM-L binding (shown in our current study and^3^,^23^) and this adhesion can promote subsequent transmigration. Whilst we do not directly demonstrate a requirement for the CAR-JAML interface in the present study, JAML is the only recognised receptor for CAR on leukocytes, strongly implying that this interaction is also required fro transmigration in our experimental system. In addition to interactions with epithelial cells, our study also points to a potential role for CAR in endothelial cells in mediating transmigration. CAR expression has been reported to be very low or absent in many human vascular endothelial cells studied to date, but CAR is expressed in bone microvascular^23^, neonatal lymphatic^16^ and cardiac endothelial tissue^34^. Expression in vessels from other origins remains unclear, but it is certainly feasible that CAR may play a role in some tissues in mediating local inflammation. This will be an interesting avenue to pursue in future studies. Our data also shows that expression of CAR in the epithelium enhances transmigration of primary neutrophils and monocytes *in vitro* and *in vivo*, as well as THP1 cells that are of monocytic origin. Whilst our current data does not distinguish between different populations of monocytes, our data strongly suggests that CAR promotes transmigration in the presence of high levels of TNFα. This would be in-line with the notion that CAR can promote transmigration of so-called classical or ‘inflammatory’ monocytes, as opposed to the non-classical ‘patrolling’ population and would further support a role for CAR in enhancing monocyte tissue infiltration during inflammatory conditions. Future experiments will focus on understanding whether CAR can also play a role in maintaining homeostatic inflammation in the lung *in vivo* using transgenic animals, as well as further investigating responses during specific lung insults such as allergens.

Our current data demonstrates that TNFR1-induces a PI-3-K/PKC signalling cascade which is a key contributing pathway leading to CAR phosphorylation and leukocyte transmigration. It is not yet clear whether this PKC-induced phosphorylation event is able to induce a conformational change in the CAR ecto-domain such that JAM-L binding is more efficient. One possibility is that phosphorylation may induce later *in cis* interactions between CAR molecules that may promote more effective JAM-L binding. Indeed a similar mechanism has been shown for JAM-L binding to CAR^23^ and may suggest that interactions between receptors both *in cis* and *in trans* must be tightly spatially and temporally regulated in order to facilitate efficient ligand binding and permit migration. It is important to note that our data shows no change in basal permeability of the epithelial layer when FL or DDCAR are overexpressed, despite a significant increase in leukocyte transmigration across these cells. This further supports a model where receptor clustering and signalling crosstalk with the junctional components and actin cytoskeleton are tightly regulated via additional chemical and physical cues. Dissecting the biophysical changes that are required for CAR function during transmigration represents an important future challenge.

In summary, our data has identified a novel mechanism in which TNFα -dependent post-translational modification of CAR mediates efficient trans-epithelial migration of leukocytes. These findings implicate CAR as an important mediator of immune cell recruitment to sites of epithelial inflammation and a new potential therapeutic target for drug design.

## Materials and Methods

### Antibodies and reagents

Anti-E-cadherin (HECD-1) antibody was from Invitrogen. Anti-CAR (H300), anti-TNFR1 (H5), anti-ICAM1 and anti-PKCδ antibodies were from Santa Cruz (Germany). Anti-phospho-PKCδ was from Cell Signalling Technology (USA). p-CAR thr290/ser293 polyclonal antibody was previously described in Morton *et al*.^15^, and was developed by Perbioscience (Thermofisher) using the peptide Ac- RTS(pT)AR(pS)YIGSNH-C and was affinity purified before use. Anti-TNFR1 blocking antibody (Mab225) was obtained from R&D Systems (USA). Anti-GFP antibody was from Roche (UK). Anti-mouse HRP and anti-rabbit-HRP were from DAKO, anti-mouse-568, anti-rabbit-568 and phalloidin-633 were all obtained from Invitrogen (UK). CalyculinA, sodium orthovanadate and PI-3-kinase inhibitor (LY294002) were obtained from Calbiochem. TNFα, IL-5, IL-1β, IL-13 and IL-17 were obtained from Sigma-Aldrich (UK). FK was produced and purified as previously described^35^. PKCδ targeted siRNA and non-targeting controls were obtained from Ambion (USA).

### Plasmids

Full length and mutant CAR sequences were cloned in frame into pHR9SIN-SEW lentiviral expression vector, which was a gift from Prof Adrian Thrasher (Institute of Child Health, UCL;^36^). Phospho-mutant CAR constructs were generated using site directed mutagenesis and have been described previously^15^. CAR shRNA vectors were in pLKO.1 backbones purchased from Sigma Mission collection (clone ID NM_001338.3-781S21C1).

### Cell culture

Immortalised human bronchial epithelial cells (HBEC) were a gift from Dr Jerry Shay (UT Southwestern^37^) and were grown in KSFM (Invitrogen). All CAR expressing stable cell lines and have been previously described^15^. 16HBE-o human bronchial epithelial cells were a gift from Prof D.Gruenert (University of Vermont, US;^38^) and were cultured in RPMI containing 10%FCS (Sigma, UK). THP-1 cells were a gift from Prof. G.E. Jones and Dr A Ivetic (King’s College London) and were maintained in RPMI supplemented with 10% FCS and 500 μM β-Mercaptoethanol. HEK293 packaging cells were used to generate lentiviral particles for viral transduction as previously described^15^.

### Confocal microscopy

Cultured cells were washed with PBS, fixed with 4% PFA in PBS for 10 min and permeabilised with 0.2% TritonX-100 for 10 min. Cells were incubated with primary antibodies for 2 hours and appropriate secondary antibodies conjugated to alexafluo-568 or cy5 and Phalloidin conjugated to Alexafluor 568 or 633 for 1 hour. Cells were mounted onto slides using Immunofluore (ICN, UK). Confocal microscopy was performed using a Nikon A1R inverted confocal laser scanning microscope with a 60x oil objective and laser excitation wavelengths of 488 nm (for GFP or Alexafluor-488), 561 nm (for Alexafluor-568) and 633 nm (for Alexafluor-633 and cy5).

For live cell imaging experiments, HBEC were plated onto glass-bottomed Ibidi chambers (Ibidi, Germany) and allowed to form an intact monolayer as determined using phase contrast microscopy. Cells were then imaged every minute using 488 nm laser excitation using a 60x oil objective on a Nikon A1R inverted confocal microscope (Nikon UK) equipped with a humidified environmental chamber heated to 37 °C, with 5%CO_2_ and PFS activated. After 30 minutes, TNF was added to the imaging media at a final concentration of 10 ng/ml and the imaging immediately resumed every minute for a further 90 minutes. All images were saved as. nd2 files and analysed in NIS Elements software (Nikon) or exported as tif files for presentation. Analysis of live cell junctional dynamics was performed on exported image sequences using ImageJ. Individual cell-cell junctions were randomly selected from the final frames of movies using a defined ROI (90 in total per condition and cell type). Using intensity linescans, junctions were classified as one of three phenotypes: ‘disassembled’: where no defined line of CAR-GFP was evident between two cells, ‘disconnected’: where an intermittent accumulation of CAR-GFP was present at the junction or ‘intact’: where a clear, dense accumulation of CAR-GFP was present as a single continuous line. Data was pooled within each independent experiment and distributions plotted as shown in Fig. 4F.

### Western blotting and immunoprecipitation

100,000 HBEC per condition were cultured either in normal growth media lysis in 100 ul sample buffer containing β-mercaptoethanol at room temperature. Lysates were immediately subjected to SDS-PAGE and blotted using nitrocellulose membrane. Blots were blocked and probed using 3% milk/PBS-0.2%tween or 5%BSA/TBS-0.1%tween. For THP-1 co-culture experiments, HBEC media was replaced with THP-1 growth media minus β-mercaptoethanol 2 hours prior to THP-1 addition. Cells were then lysed and analysed as before. For IP experiments, CAR-GFP HBEC were cultured in growth media for 48 hours before treatment with CalA and lysis in IP lysis buffer (pH7.4 50 mM Tris, 150 mM NaCl, 1 mM EDTA, 1% Triton, 1% NP40, PI cocktail). Lysates were incubated with 5μg anti-GFP antibody pre-bound to A/G agarose beads overnight before washing the beads with 1 ml IP lysis buffer 3 times. Immunocomplexes were separated using SDS-PAGE and immunoblotted for specified proteins.

### Trans-epithelial migration assays

HBEC or 16HBE14-o cells were seeded in 6.5 mm Transwell chambers (Corning, UK) with 8.0 μm pores at 30,000 cells per chamber and allowed to form monolayers. Media in the upper and lower chambers was changed to RPMI containing 10% FBS before addition of 100,000 THP-1 cells, primary monocytes or primary neutrophils stained with Cell Tracker^TM^ Orange Dye (Molecular probes, UK) were added to the top well of the chambers. After 24 hours, the number of THP-1 cells in the lower chamber was counted using a FACSCalibur flow cytometer (BD Biosciences). For Ad5 fibre-knob competition assays recombinant Ad5FK or BSA control was added to HBEC cells 3 hours after seeding and remained in the media throughout the experiment. Data is presented as percentage of total cells added that underwent transmigration.

### Inverted trans-epithelial migration assays

HBEC or 16HBE14-o cells were seeded in 6.5 mm Transwell chambers (Corning, UK) with 8.0μm pores at 30,000 cells per chamber and allowed to form monolayers. Transwell inserts were then inverted and 100,000 THP-1 stained with Cell Tracker^TM^ Orange Dye (Molecular probes, UK) were added to the underside of the Transwells. The THP-1 cells were allowed to adhere for 1 hr before placing them back into the plate. To encourage TEpM against gravity RPMI containing 1% FBS as place into the lower chamber while RPMI containing 10% FBS was place in the upper chamber. After 24 hours the Transwell inserts were removed, fixed in PFA and stained with phalloidin. A total of 3 5 × 5 tile-scans per plane (basal surface, within the monolayer and apical surface) per sample were obtained using a 40x air objective on a Nikon A1R inverted laser scanning confocal microscope. The tiles were assembled together using Nikon NIS Elements software and further analysed to count the number of THP-1 present within each level. Data presented is only THP-1 present on the apical surface of the HBEC monolayer. Data is presented as percentage of total cells added that underwent transmigration.

### Isolation of primary human monocytes and neutrophils

Citrated blood (1 ml citrate/10 ml of blood) was collected from healthy volunteers in accordance with the locally approved ethical guidelines and following written informed consent from all subjects. Procedures were approved by Guy’s and St Thomas’ NHS Foundation Trust and King’s College London. Blood was gently inverted and diluted with equal quantity of MACS buffer (50 ml of 2% FBS-PBS and 0.2 ml of 0.5M of EDTA). The diluted blood was gently layered above 15 ml of Lymphoprep (Stemcell Technologies, USA) and after centrifugation (800 g, 20 min, without break) peripheral blood mononuclear cells (PBMCs) were collected and washed. Monocytes were negatively isolated from the PBMC population using the Monocyte Isolation Kit II (Miltenyi Biotec, USA) following manufacturer´s instructions. Evaluation of monocyte purity was achieved by incubating the cells for 30 minutes on ice with a viability dye (Live/dead Fixable Aqua, Life Technologies) followed by a second incubation using monoclonal antibodies: CD14-APC (61D3), CD16-PE (eBioCB16) from eBioscience. Streptavidin-PE/Cy5 (BD Pharmingen, UK) was added to check the possible contaminant cells. The acquisition was performed using an Attune NxT flow cytometer (Life Technologies) and analysed with FlowJo v9.8 software (FlowJo, LLC). Neutrophils were isolated from freshly drawn anti-coagulated whole blood from healthy volunteers using the MACSxpress^®^ Neutrophils Isolation Kit from Milteny Biotec following manufacturer´s instructions. Removal of residual erythrocytes was done using a Red Blood Cell Lysis Solution (Milteny Biotec, USA) and evaluation of neutrophil purity was performed using CD15-FITC (HI98), CD16-PE (eBioCB16) both from eBioscience and Streptavidin-PE/Cy5 (BD Pharmingen, UK) followed by acquisition in a Attune NxT flow cytometer (Life Technologies). A viability dye-Aqua (Life Technologies) was used in a previous incubation to exclude the dead cells. Analysis was performed using FlowJo v9.8 (FlowJo, LLC) software. Transmigration assays were performed as described for THP-1 cells.

### THP-1 adhesion assays

For HBEC adhesion assays, HBEC were seeded onto 13 mm coverslips and allowed to form monolayers. After 48 hours 100,000 THP-1 cells stained with Cell Tracker^TM^ Orange (Molecular Probes, UK) were added to the monolayers in RPMI supplemented with 10% FBS. THP-1 cells were allowed to adhere to HBEC monolayers for 24 hours before fixation and immunostaining with Phalloidin-633. A total of 5 5 × 5 tile-scans per sample were obtained using a 40x air objective on a Nikon A1R confocal microscope. The tiles were assembled together using Nikon NIS Elements software and further analysed using Cell Profiler (BD, UK) to count the number of adhered THP-1 cells per image.

### THP-1- integration assay

HBEC were seeded onto collagen coated 12 well plates and allowed to form monolayers over 3–4 days. THP-1 were stained with Cell Tracker^TM^ Orange (Molecular Probes, UK) before adding to the monolayers in RPMI supplemented with 10% FBS and fixation with formaldehyde (final concentration 4%) added directly to the medium 4 or 6 hours after addition of THP-1. Where appropriate, HBEC monolayers were incubated with 10 ng/ml TNFα that was removed prior to addition of THP-1. For TNFR1 inhibitor experiments, TNFR1 blocking antibody (Mab225, R&D Systems, UK) was added to HBEC monolayers for 30 min prior to removal of excess antibody and addition of THP-1 cells. Phase and red channel images were acquired using a 20x air objective on an Olympus IX71 inverted widefield microscope. 5 images were taken per well. Adherent THP-1 were identified using the red channel image and analysed as phase dark (integrated into the monolayer) and phase bright (not integrated). Data from 5 images per condition were averaged per experiment and presented as mean + /− SEM. Experiments were repeated 3 times and a representative data set is shown.

### FITC-dextran permeability assays

Cells were seeded in 6.5 mm collagen coated Transwell chambers (Corning) with 8.0 μm pores at 100,000 cells per well. Wells were reviewed after 24 hrs to ensure an even covering of cells with a stable complete monolayer. TNFα was added to the specific treatment wells, followed by 10 μl of FITC-dextran (20 KDa; Sigma) solution added to the upper chamber of all wells. 100 μl of the media was then collected from the lower chamber at 30 minutes and one hour after addition of FITC-dextran. The collected samples were then compared for relative FITC-dextran concentration on a Fluostar Omega fluorescence plate reader (BMG). The system compared light absorption from each sample to provide a comparative reading of light transmission between the samples.

### Acute inflammation mouse model

C57BL/6 (B6) mice (Harlan) were used at 4–8 weeks. All experiments were approved by King’s College London animal welfare committee and performed under UK Home Office Regulations. For mucosal sensitization 1 μg recombinant murine TNF-α (Immunotools) was given intranasally in 50 μl PBS/mouse under light inhaled anaesthesia (isoflurane). After 24 hours animals were killed and bronchoalveolar lavage (BAL) performed using 1 ml PBS. Flow cytometry. Inflammatory cells in BAL were identified as described in (van Rijt *et al*., 2004) with addition of anti-Gr-1 to identify neutrophils (Gr-1 + CD11c-CCR3-CD4-CD8-B220-). Antibody staining (0.1 μg/sample, all eBioscience) was performed in PBS 1% FCS with each BAL sample after washing and analyzed on a FACScalibur (BD Bioscience, UK). Total cell numbers were calculated by analysis of fixed sample volumes, validated with fluorescent beads.

### Chronic inflammation mouse model

For the chronic inflammatory mouse model paraffin embedded slides of mouse lung sections were generated from BALB/c 6-wk-old mice, sensitized by i.p. injection of 10 μg OVA diluted 1:1 with adjuvant, followed by a second sensitization on day 12. At day 19, mice were challenged daily by oropharyngeal administration of either 400 μg/ml OVA in 50 μl saline or 50 μl saline alone for 6 d, followed by additional challenges on days 26, 28, 30, and 33. The mice were sacrificed on day 34. For formalin-fixed tissue, the left lobe was inflated with formalin and fixed in formalin overnight and then embedded in paraffin wax. The slides were initially heated at 75 °C to warm the paraffin. The slides were then rehydrated via a step wise process of 2x immersion in xylene (supplied by Fisher scientific) for 10 minutes followed by 2 x immersion in 100% ethanol, 1x immersion in 90% ethanol, 1x immersion in 70% ethanol, 1 immersion in 50% ethanol all for 5 mins each. Antigen retrieval was then performed in a citrate buffer at a pH of 6 in a pressure cooker for 15 minutes.

### TNFR1/2 knockout mouse model

TNFR1/2KO mice were generated in the C57BL/6 background and have been previously published^39^. For the TNFR1/2KO model paraffin embedded slides of mouse lung sections were generated from C57BL/6 WT and TNFR1/2 KOmice, sensitized by i.p. injection of 10 μg OVA in 150 μl PBS combined 1:1 with Complete Freunds Adjuvant (containing 1 mg/ml bacterial load), followed by a second sensitization on day 12 this time using Incomplete Freunds Adjuvant (without bacterial load). At day 19, mice were challenged with intra-nasal administration of either 10 μg/ml OVA in 50 μl saline or 50 μl saline alone, followed by additional challenges on days 21, 23, and 24. The mice were sacrificed on day 34. For formalin-fixed tissue, the left lobe was inflated with formalin and fixed in formalin overnight and then embedded in paraffin wax. Slides were then treated as stated earlier. On lobe was frozen at −80 °C for processing by western blot.

### Statistical analysis

Results are expressed as the mean ± SEM from the specified number of experiments, as indicated in the figure legends. Student’s t-test or ANOVA were used to analyse statistical significance as appropriate.

## Additional Information

**How to cite this article**: Morton, P. E. *et al*. TNFα promotes CAR-dependent migration of leukocytes across epithelial monolayers. *Sci. Rep.* **6**, 26321; doi: 10.1038/srep26321 (2016).

## Supplementary Material

Supplementary Information

## References

[b1] Davies D. E. The role of the epithelium in airway remodeling in asthma. Proc Am Thorac Soc 6, 678–682 (2009). 20008875 10.1513/pats.200907-067DP PMC2797070

[b2] Tam A., Wadsworth S., Dorscheid D., Man S. F. & Sin D. D. The airway epithelium: more than just a structural barrier. Ther Adv Respir Dis 5, 255–273 (2011). 21372121 10.1177/1753465810396539

[b3] Zen K. . Neutrophil migration across tight junctions is mediated by adhesive interactions between epithelial coxsackie and adenovirus receptor and a junctional adhesion molecule-like protein on neutrophils. Mol Biol Cell 16, 2694–2703 (2005). 15800062 10.1091/mbc.E05-01-0036 PMC1142417

[b4] Zen K. & Parkos C. A. Leukocyte-epithelial interactions. Curr Opin Cell Biol 15, 557–564 (2003). 14519390 10.1016/s0955-0674(03)00103-0

[b5] Coyne C. B. . Regulation of airway tight junctions by proinflammatory cytokines. Mol Biol Cell 13, 3218–3234 (2002). 12221127 10.1091/mbc.E02-03-0134 PMC124154

[b6] Gonzalez-Mariscal L., Tapia R. & Chamorro D. Crosstalk of tight junction components with signaling pathways. Biochim Biophys Acta 1778, 729–756 (2008). 17950242 10.1016/j.bbamem.2007.08.018

[b7] Luissint A. C., Nusrat A. & Parkos C. A. JAM-related proteins in mucosal homeostasis and inflammation. Semin Immunopathol 36, 211–226 (2014). 24667924 10.1007/s00281-014-0421-0 PMC4084508

[b8] Xiao C. . Defective epithelial barrier function in asthma. J Allergy Clin Immunol 128, 549–556 e541-512 (2011). 10.1016/j.jaci.2011.05.038 21752437

[b9] Bergelson J. M. . Isolation of a common receptor for Coxsackie B viruses and adenoviruses 2 and 5. Science 275, 1320–1323 (1997). 9036860 10.1126/science.275.5304.1320

[b10] Cohen C. J. . The coxsackievirus and adenovirus receptor is a transmembrane component of the tight junction. Proc Natl Acad Sci USA 98, 15191–15196 (2001). 11734628 10.1073/pnas.261452898 PMC65005

[b11] Raschperger E. . The coxsackie- and adenovirus receptor (CAR) is an *in vivo* marker for epithelial tight junctions, with a potential role in regulating permeability and tissue homeostasis. Exp Cell Res 312, 1566–1580 (2006). 16542650 10.1016/j.yexcr.2006.01.025

[b12] Schmitz H. . Altered tight junction structure contributes to the impaired epithelial barrier function in ulcerative colitis. Gastroenterology 116, 301–309 (1999). 9922310 10.1016/s0016-5085(99)70126-5

[b13] Coyne C. B. & Bergelson J. M. CAR: a virus receptor within the tight junction. Adv Drug Deliv Rev 57, 869–882 (2005). 15820557 10.1016/j.addr.2005.01.007

[b14] Hussain F. . CAR modulates E-cadherin dynamics in the presence of adenovirus type 5. PLoS One 6, e23056 (2011). 21850251 10.1371/journal.pone.0023056 PMC3151283

[b15] Morton P. E., Hicks A., Nastos T., Santis G. & Parsons M. CAR regulates epithelial cell junction stability through control of E-cadherin trafficking. Sci Rep 3, 2889 (2013). 24096322 10.1038/srep02889 PMC3791454

[b16] Mirza M. . Essential role of the coxsackie- and adenovirus receptor (CAR) in development of the lymphatic system in mice. PLoS One 7, e37523 (2012). 22624044 10.1371/journal.pone.0037523 PMC3356332

[b17] Pazirandeh A. . Multiple phenotypes in adult mice following inactivation of the Coxsackievirus and Adenovirus Receptor (Car) gene. PLoS One 6, e20203 (2011). 21674029 10.1371/journal.pone.0020203 PMC3108585

[b18] Verdino P., Witherden D. A., Havran W. L. & Wilson I. A. The molecular interaction of CAR and JAML recruits the central cell signal transducer PI3K. Science 329, 1210–1214 (2010). 20813955 10.1126/science.1187996 PMC2951132

[b19] Weber D. A. . Neutrophil-derived JAML inhibits repair of intestinal epithelial injury during acute inflammation. Mucosal Immunol 7, 1221–1232 (2014). 24621992 10.1038/mi.2014.12 PMC4340686

[b20] Witherden D. A. . The junctional adhesion molecule JAML is a costimulatory receptor for epithelial gammadelta T cell activation. Science 329, 1205–1210 (2010). 20813954 10.1126/science.1192698 PMC2943937

[b21] Guo Y. L. . Role of junctional adhesion molecule-like protein in mediating monocyte transendothelial migration. Arterioscler Thromb Vasc Biol 29, 75–83 (2009). 18948633 10.1161/ATVBAHA.108.177717

[b22] Kirby I. . Identification of contact residues and definition of the CAR-binding site of adenovirus type 5 fiber protein. J Virol 74, 2804–2813 (2000). 10684297 10.1128/jvi.74.6.2804-2813.2000 PMC111771

[b23] Luissint A. C., Lutz P. G., Calderwood D. A., Couraud P. O. & Bourdoulous S. JAM-L-mediated leukocyte adhesion to endothelial cells is regulated in cis by alpha4beta1 integrin activation. J Cell Biol 183, 1159–1173 (2008). 19064666 10.1083/jcb.200805061 PMC2600739

[b24] Cain R. J., Vanhaesebroeck B. & Ridley A. J. The PI3K p110alpha isoform regulates endothelial adherens junctions via Pyk2 and Rac1. J Cell Biol 188, 863–876 (2010). 20308428 10.1083/jcb.200907135 PMC2845076

[b25] Schulzke J. D. . Epithelial tight junctions in intestinal inflammation. Ann N Y Acad Sci 1165, 294–300 (2009). 19538319 10.1111/j.1749-6632.2009.04062.x

[b26] McKenzie J. A. & Ridley A. J. Roles of Rho/ROCK and MLCK in TNF-alpha-induced changes in endothelial morphology and permeability. J Cell Physiol 213, 221–228 (2007). 17476691 10.1002/jcp.21114

[b27] Mazzon E. & Cuzzocrea S. Role of TNF-alpha in lung tight junction alteration in mouse model of acute lung inflammation. Respir Res 8, 75 (2007). 17971210 10.1186/1465-9921-8-75 PMC2174464

[b28] Al-Sadi R. . Mechanism of interleukin-1beta induced-increase in mouse intestinal permeability *in vivo*. J Interferon Cytokine Res 32, 474–484 (2012). 22817402 10.1089/jir.2012.0031 PMC3464071

[b29] Turner J. R. Intestinal mucosal barrier function in health and disease. Nat Rev Immunol 9, 799–809 (2009). 19855405 10.1038/nri2653

[b30] Satsu H. . Induction by activated macrophage-like THP-1 cells of apoptotic and necrotic cell death in intestinal epithelial Caco-2 monolayers via tumor necrosis factor-alpha. Exp Cell Res 312, 3909–3919 (2006). 17010338 10.1016/j.yexcr.2006.08.018

[b31] Coyne C. B., Voelker T., Pichla S. L. & Bergelson J. M. The coxsackievirus and adenovirus receptor interacts with the multi-PDZ domain protein-1 (MUPP-1) within the tight junction. J Biol Chem 279, 48079–48084 (2004). 15364909 10.1074/jbc.M409061200

[b32] Excoffon K. J., Hruska-Hageman A., Klotz M., Traver G. L. & Zabner J. A role for the PDZ-binding domain of the coxsackie B virus and adenovirus receptor (CAR) in cell adhesion and growth. J Cell Sci 117, 4401–4409 (2004). 15304526 10.1242/jcs.01300

[b33] Erle D. J. & Sheppard D. The cell biology of asthma. J Cell Biol 205, 621–631 (2014). 24914235 10.1083/jcb.201401050 PMC4050726

[b34] Kaur T. . Expression of coxsackievirus and adenovirus receptor and its cellular localization in myocardial tissues of dilated cardiomyopathy. Exp Clin Cardiol 17, 183–186 (2012). 23592932 PMC3627271

[b35] Kirby I. . Mutations in the DG loop of adenovirus type 5 fiber knob protein abolish high-affinity binding to its cellular receptor CAR. J Virol 73, 9508–9514 (1999). 10516059 10.1128/jvi.73.11.9508-9514.1999 PMC112985

[b36] Demaison C. . High-level transduction and gene expression in hematopoietic repopulating cells using a human immunodeficiency [correction of imunodeficiency] virus type 1-based lentiviral vector containing an internal spleen focus forming virus promoter. Hum Gene Ther 13, 803–813 (2002). 11975847 10.1089/10430340252898984

[b37] Ramirez R. D. . Immortalization of human bronchial epithelial cells in the absence of viral oncoproteins. Cancer Res 64, 9027–9034 (2004). 15604268 10.1158/0008-5472.CAN-04-3703

[b38] Gruenert D. C., Basbaum C. B. & Widdicombe J. H. Long-term culture of normal and cystic fibrosis epithelial cells grown under serum-free conditions. In Vitro Cell Dev Biol 26, 411–418 (1990). 1693142 10.1007/BF02623833

[b39] Finsterbusch M., Voisin M. B., Beyrau M., Williams T. J. & Nourshargh S. Neutrophils recruited by chemoattractants in vivo induce microvascular plasma protein leakage through secretion of TNF. J Exp Med 211, 1307–1314 (2014). 24913232 10.1084/jem.20132413 PMC4076577

