# Supplementary material for: TNFα promotes CAR-dependent migration of leukocytes across epithelial monolayers
Source: Sci Rep. 2016 May 19;6:26321. doi: 10.1038/srep26321 (PMC4872059; doi:10.1038/srep26321)
Supplement: Supplementary Information [file srep26321-s1.pdf]

Supplementary information

TNFα promotes CAR-dependent migration of leukocytes across epithelial monolayers

Penny E. Morton[1,6], Alexander Hicks[2,6], Elena Ortiz-Zapater[2], Swetavalli Raghavan[1], Rosemary Pike[1], Alistair Noble[2], Abigail Woodfin[3], Gisli Jenkins[4], Emma Rayner[5], George Santis[2,6], Maddy Parsons[1,6,7]

1. Randall Division of Cell and Molecular Biophysics, King's College London, New Hunt's House, Guys Campus, London, UK, SE1 1UL
2. Division of Asthma, Allergy & Lung Biology, King's College London, 5th Floor Tower Wing, Guy's Hospital Campus, London, UK, SE1 1UL
3. William Harvey Research Institute, Barts and the London School of Medicine and Dentistry, Queen Mary, University of London, EC1M 6BQ
4. Respiratory Research Unit, University of Nottingham, Clinical Sciences Building, City Campus, Nottingham, UK, NG5 1PB
5. Public Health England, Salisbury, Wiltshire, UK, SP4 0JG
6. These authors contributed equally to the study
7. To whom correspondence should be addressed: maddy.parsons@kcl.ac.uk

Supplementary Figure 1

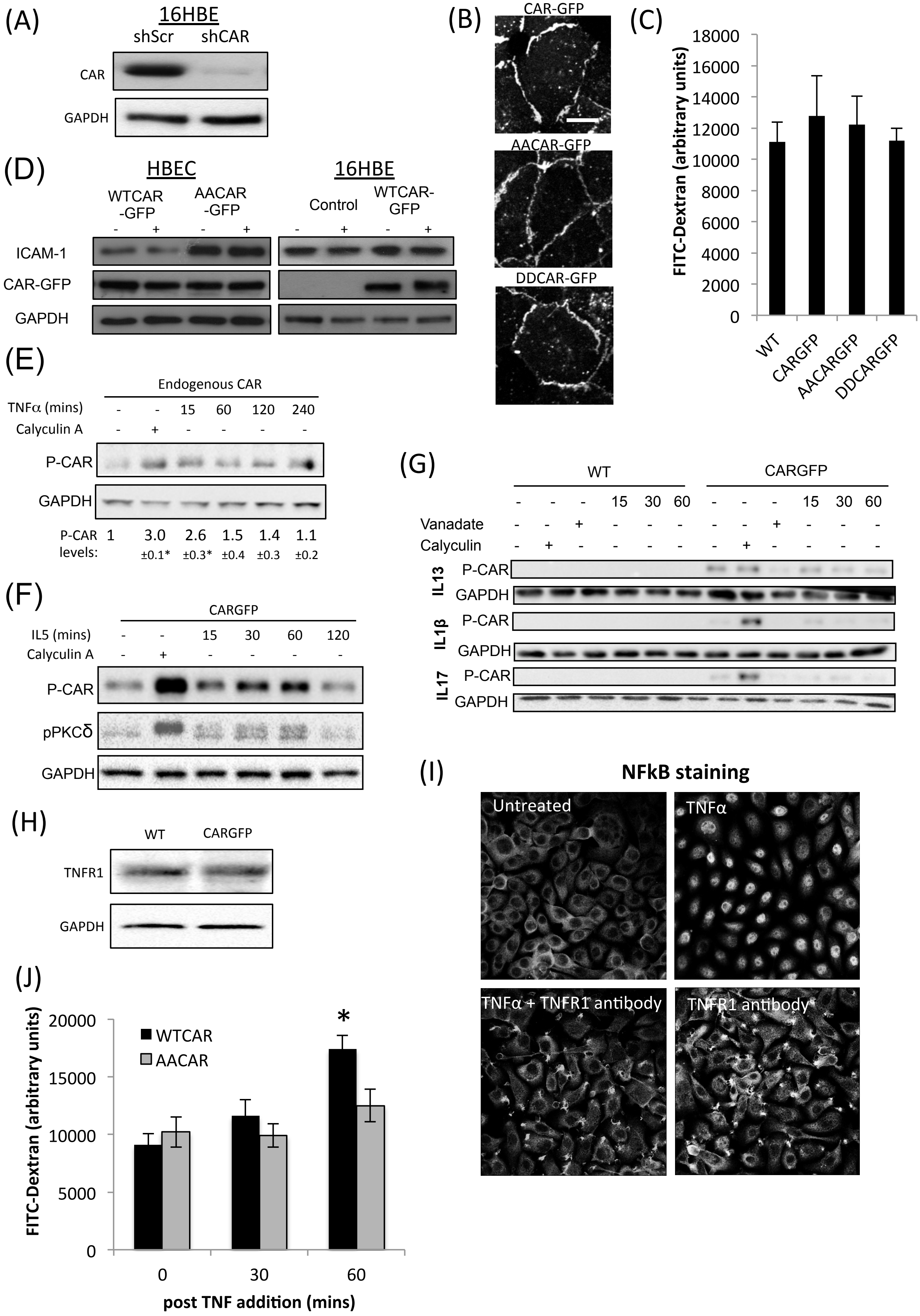

Supplementary Figure 2

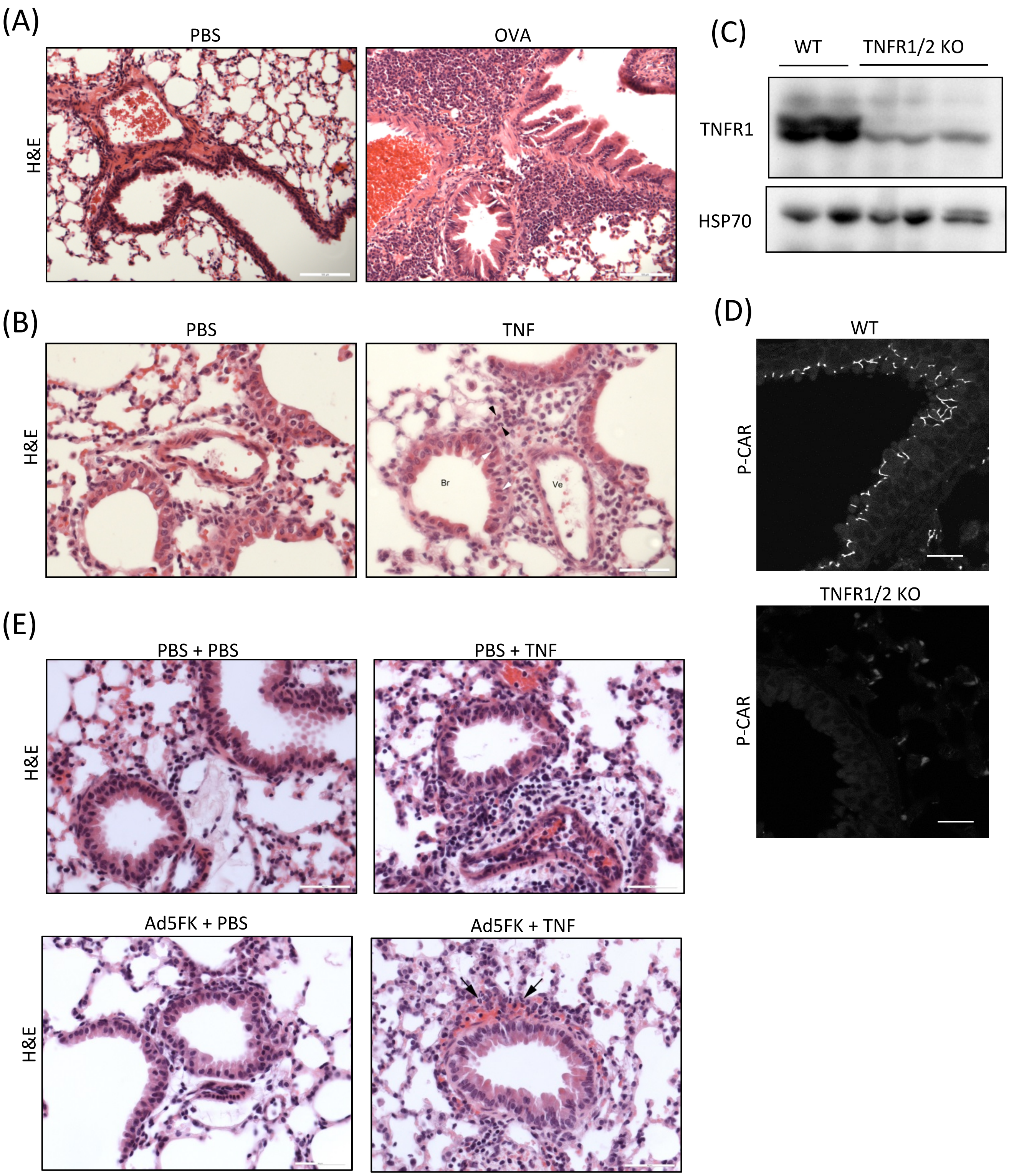

Supplementary figure legends

Supplementary Figure 1:
(A) Representative western blot of CAR levels in 16HBE cells infected with control shRNA (shScr) or an shRNA targeted to CAR (shCAR). GAPDH serves as a loading control. (B) Representative confocal images of CAR-GFP localisation in HBEC stably expressing FLCAAR-GFP, AACAR-GFP or DDCAR-GFP. GFP channel is shown. Scale bar is 10µm. (C) Graph of cell permeability assays as determined by FITC dextran movement across intact monolayers of specified HBEC cultures in Transwells. Values shown are mean intensity levels of FITC dextran measured on a spectrophotometer (arbitrary units) pooled from 4 wells per cells line +/-SEM. Representative of 4 independent experiments. (D) Representative westerns blots of ICAM1 and CAR-GFP levels in specified HBEC or 16HBE cells with or without TNFα treatment for 1 hour. GAPDH serves as a loading control. (E) Western blot of lysates from 16HBE cells treated with TNFa for specified times, lysed and probed for endogenous phospho-CAR and GAPDH as a loading control. Calyculin A was used as a positive control to induce phosphorylation. Representative of 3 independent experiments. (F) Analysis of IL-5 driven phospho-CAR and phospho-PKCδ by western blot. CAR-GFP HBEC were treated with 25ng/ml IL-5 for the times indicated and lysed. Western blots were probed for phospho-CAR, phospho-PKCδ and GAPDH as a loading control (G) Western blots analysis of CAR phosphorylation and GADPH (loading control) after treatment with IL-13, IL-7 or IL-1β for the indicated times. (H) Western blot analysis of TNFR1 expression levels in WT and CARGFP HBEC. Western blots were probed with anti-TNFR1 antibody (H5) and HSC70 as a loading control. (I) Confocal analysis of NFκB immuno-stained WT or CAR-GFP HBEC. Cells were treated with 10ng/ml TNFα and 10µg/ml TNFR1 blocking antibody where indicated. (J) Graph of cell permeability assays as determined by FITC dextran movement across intact monolayers of specified HBEC cultures in Transwells in the presence or absence of TNFα over stated time periods. Values shown are mean intensity levels of FITC dextran measured on a spectrophotometer (arbitrary units) pooled from 4 wells per cells line +/-SEM. Representative of 3 independent experiments. *denotes $p<0.01$ compared to equivalent cells at time 0.

Supplementary Figure 2:
(A) Photomicrographs of H&E stained lung tissue used in 4A. Calibration bar=100µm. (B) Photomicrographs of lung tissue used in B. Ve=vessel; Br=bronchiole; arrow heads point to neutrophils. Calibration bar=50µm. HE. (C) Western blot analysis of phospho-CAR in lysates from lungs of WT or TNFR1/2 KO mice sensitized with Ovalbumin. Western blots were probed for phospho-CAR and HSP70 as a loading control. (D) Example confocal images of lung sections from WT (top panel) and TNFR1/2 KO (bottom panel) mice sensitized with Ovalbumin and immunostained for phospho-CAR. Scale bar corresponds to 20µm. (E) Photomicrographs of H&E stained lung tissue used in 4E. Arrows show infiltrating neutrophils. Scale bar is 50 µm.
